# Warm Deformation Behavior and Flow Stress Modeling of AZ31B Magnesium Alloy under Tensile Deformation

**DOI:** 10.3390/ma16145088

**Published:** 2023-07-19

**Authors:** Mohanraj Murugesan, Jae-Hyeong Yu, Wanjin Chung, Chang-Whan Lee

**Affiliations:** 1 Department of Mechanical System Design Engineering, Seoul National University of Science and Technology, Seoul 01811, Republic of Korea; mohanaero45@seoultech.ac.kr (M.M.); wjchung@seoultech.ac.kr (W.C.); 2 Department of Mechanical Information Engineering, Seoul National University of Science and Technology, Seoul 01811, Republic of Korea; jhyu9109@seoultech.ac.kr

**Keywords:** AZ31 magnesium alloy, warm deformation, constitutive equation, flow stress, original Johnson–Cook model, modified JC model, modified Zerilli–Armstrong model

## Abstract

Constitutive equations were recognized for AZ31B magnesium alloy at higher temperatures and strain rates from conventional empirical models like the original Johnson–Cook (JC), modified JC, and modified Zerilli–Armstrong (ZA) models for capturing the material warm deformation behavior. Uniaxial warm tensile tests were performed at temperatures (50 to 250 °C) and strain rates (0.005 to 0.0167 s${}^{-1}$) to probe AZ31 magnesium alloy flow stress values. Depending on the calculated flow stress, constitutive equations were recognized, and these established models were assessed by the coefficient of determination ($R^{2}$), relative mean square error (RMSE), and average absolute relative error (AARE) metrics. The results demonstrated that the flow stress calculated by the modified JC and ZA models revealed good agreement against the test data. Thus, the outcomes confirmed that the recognized modified JC and modified ZA models could effectively forecast AZ31 magnesium alloy flow behavior by capturing the material deformation behavior accurately.

## 1. Introduction

Magnesium alloys are widely used in industries due to their lightweight properties and desirable characteristics like low density, dimensional stability, specific strength, damping capacity, thermal conductivity, electromagnetic shielding, and recyclability potential [1,2]. However, at room temperature, their material formability is restrained because of their hexagonal close-packed structure, primarily allowing basal slip activation [2,3]. This limitation hinders their further development and application. To enhance the plastic formability, it is crucial to review magnesium alloy flow stress behavior under hot deformation and understand their deformation characteristics. Theoretical constitutive models are commonly employed in material processing to forecast behavior, considering work hardening, strain-rate sensitivity, and thermal softening under various conditions [3,4,5]. The accurate modeling of ductile materials is vital for effective numerical simulations using finite element (FE) tools, as this enables the simulation of their thermal–mechanical response. Developing constitutive models involves systematically determining parameters by fitting calculated stress values. This approach allows for the precise characterization of material behavior. A well-designed and reliable flow stress model plays a significant role in predicting material ductility across various strain rates and temperatures, thereby facilitating efficient product design. Although constitutive models are divided into different types, their ultimate goal remains the precise representation of material behavior [6,7].

Numerous constitutive models have been proposed to forecast material deformation behavior. One notable example is the Johnson–Cook (JC) flow stress model, introduced by Johnson and Cook, specifically tailored to capture the behavior of ductile materials under large deformation conditions [8]. Zhang et al. [9] utilized the JC model to examine the hard turning process and achieved better agreement with experimental observations. Milani et al. [10] employed an optimization procedure to determine JC material coefficients for two alloy materials, namely, super alloy and titanium, reducing the number of experiments and obtaining commendable results. Banerjee et al. [11] and Buzyurkin et al. [12] studied armor steel and titanium alloys, respectively, using the JC model, improving agreement between simulations and experiments. Majzoobi et al. [13] employed optimization techniques to estimate JC material and damage model coefficients, showing better similarity with test and numerical predictions. Aviral Shrot et al. [14] recommended a procedure utilizing the Levenberg–Marquardt (LM) algorithm for JC material coefficient identification, successfully estimating these parameters for a machining process. However, it is important to note that the original JC model lacks the consideration of coupled effects, which may reduce prediction accuracy for material flow stress values.

Researchers have recently focused on improving model predictability through precise constitutive models. These models are essential for optimizing forming processes and establishing reliable finite element (FE) models. Hongyi et al. [15] investigated the flow stress of a beta titanium material with the original JC and modified Zerilli-Armstrong (ZA) models, finding precise correlation with the test data using the modified ZA model. Guang et al. [16] employed a modified JC model to forecast deformation behavior in aluminum alloy material, effectively explaining strain hardening and thermal softening characteristics compared to actual flow stress data. Uma et al. [17] recommended a constitutive equation for aluminum alloy material from modified JC and ZA models, yielding satisfactory flow stress predictions from both models. However, the JC material model did not align well with test data. Dipti et al. [18] utilized strain-compensated Arrhenius-type equation, modified JC, and ZA models for modified steel material, reporting good agreement between proposed models and the test observations. The strain-compensated constitutive equation demonstrated more precise tracking of deformation behavior compared to other models.

Liu et al. [19] derived a new flow stress equation for AZ31B magnesium alloy, incorporating dynamic recrystallization. The proposed model exhibited excellent agreement with experimental data, showing higher prediction accuracy. Cheng et al. [20] used the Fields–Backofen constitutive equation with a softening term added to the work-hardening stage, significantly improving flow stress prediction during softening. Nguyen et al. [21] proposed a constitutive model considering both strain-hardening and softening stages for AZ31B magnesium alloy material deformation, leading to better flow stress prediction and good comparison with the test data. Luan et al. [22] recommended a constitutive equation for AZ31B magnesium alloy, adjusting measured flow curves through the correction of friction and temperature. The calculated flow stress matched with the adjusted stress, demonstrating the effectiveness and accuracy of the proposed model. Despite significant research, further investigation is needed to understand the AZ31B magnesium alloy warm tensile deformation behavior, assess material workability, and optimize hot sheet forming process parameters [23]. This is crucial for achieving significant prediction accuracy and extensive applicability.

This study aims to identify an appropriate constitutive equation for modeling AZ31 magnesium alloy hot deformation behavior across a wide range of conditions, using flow curves obtained from warm tensile tests. The researchers conducted uniaxial isothermal warm tensile tests on AZ31 magnesium alloy sheets along three rolling directions: 0°, 45°, and 90° under different temperatures (50 to 250 °C) and strain rates (0.005 to 0.0167 s${}^{-1}$). Depending on the calculated stress values, constitutive models were developed using conventional flow stress models like the original JC, modified JC, and modified ZA models. The performance of these proposed models was then evaluated using metrics such as the coefficient of determination ($R^{2}$), relative mean square error (RMSE), and average absolute relative error (AARE).

## 2. Experimental Procedures

**Figure 1:**
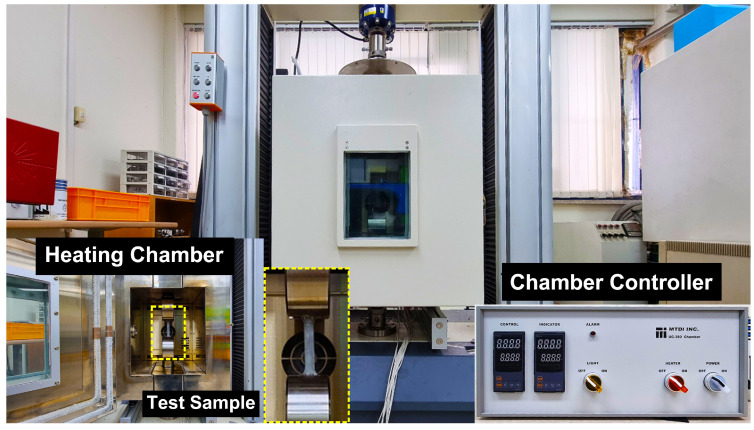
Experimental setup adopted for performing the warm tensile tests.

**Figure 2:**
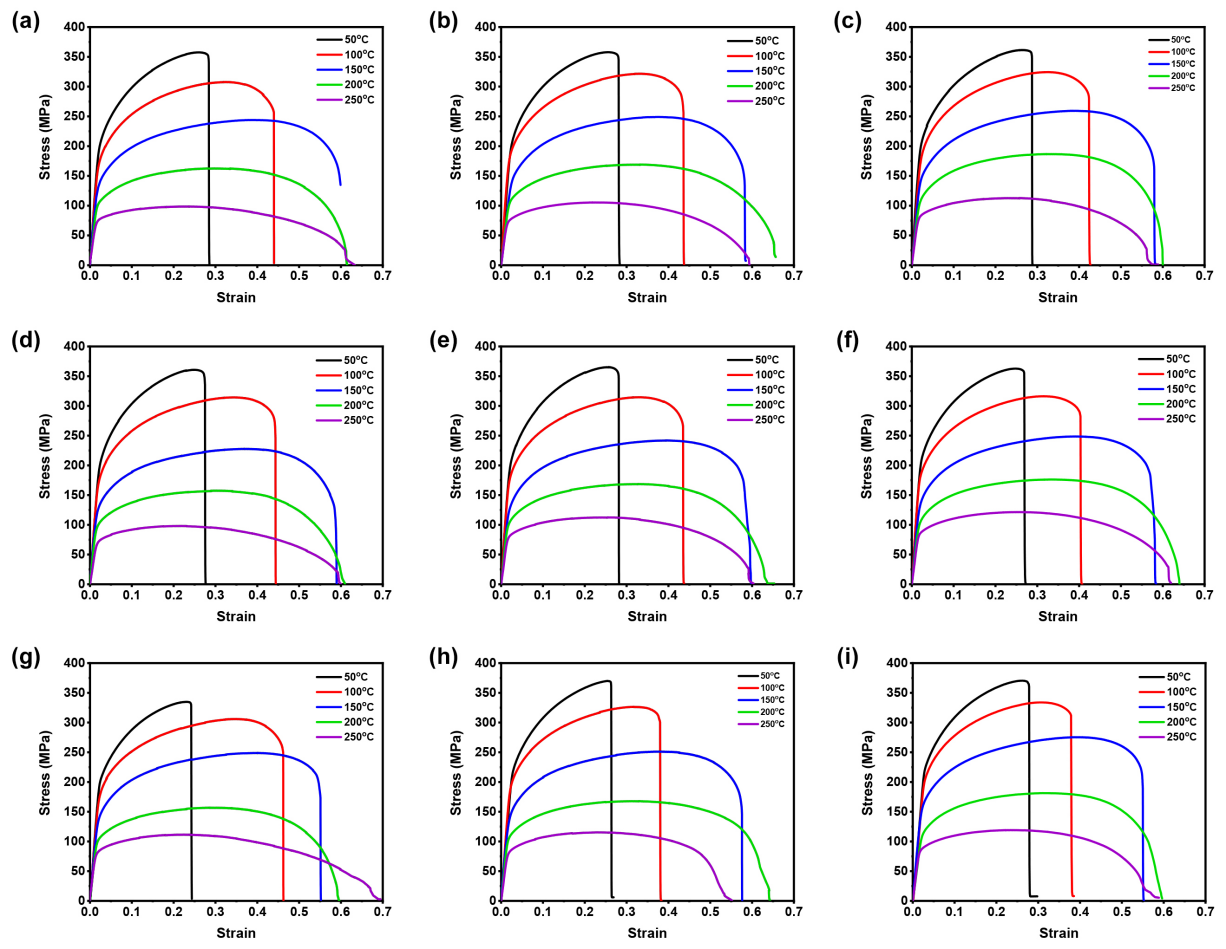
True strain–true stress data obtained from hot tensile tests. (**a**) 0.005 s${}^{-1}$; (**b**) 0.01 s${}^{-1}$; (**c**) 0.0167 s${}^{-1}$ at 0° RD. (**d**) 0.005 s${}^{-1}$; (**e**) 0.01 s${}^{-1}$; (**f**) 0.0167 s${}^{-1}$ at 45° RD. (**g**) 0.005 s${}^{-1}$; (**h**) 0.01 s${}^{-1}$; (**i**) 0.0167 s${}^{-1}$ at 90° RD.

The AZ31B magnesium alloy material was used for investigation and the chemical compositions were as follows (in wt.%): (bal.) Mg, 2.50–3.50Al, 0.60–1.40Zn, 0.20Mn, 0.10Si, 0.050Cu, 0.040Ca, 0.005Fe, and 0.005Ni [24]. The test samples of 6 mm in width and 12 mm gauge length were prepared from 1 mm thick AZ31B magnesium alloy sheets based on ASTM-E8M subsize standard with three rolling directions (RD’s), such as 0°, 45°, and 90°, for conducting the warm tensile experiments under different temperatures (50 to 250 °C) and strain rates (0.005 to 0.0167 s${}^{-1}$), as shown in Figure 1. From Figure 1, it can be observed that the isolation chamber was used to accomplish isothermal conditions. Further, the specimens were tested, and load vs. displacement data were obtained from a tensile testing machine. The stress-strain data were then averaged and converted into true flow curves with the help of standard equations [25]. Figure 2 illustrates the true flow curves received from the warm tensile tests of AZ31B magnesium alloy material under different deformation conditions with respect to three rolling directions.

**Figure 3:**
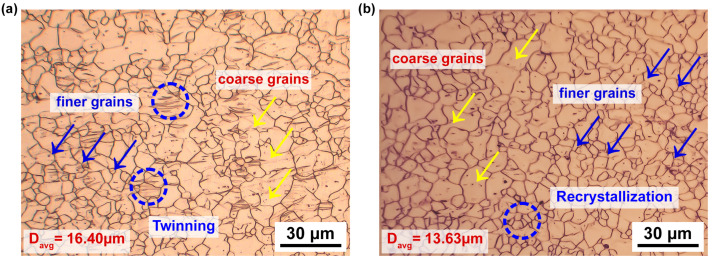
Microstructure of AZ31B magnesium alloy at 30 μm scale (**a**) 25 °C and (**b**) 250 °C.

**Figure 4:**
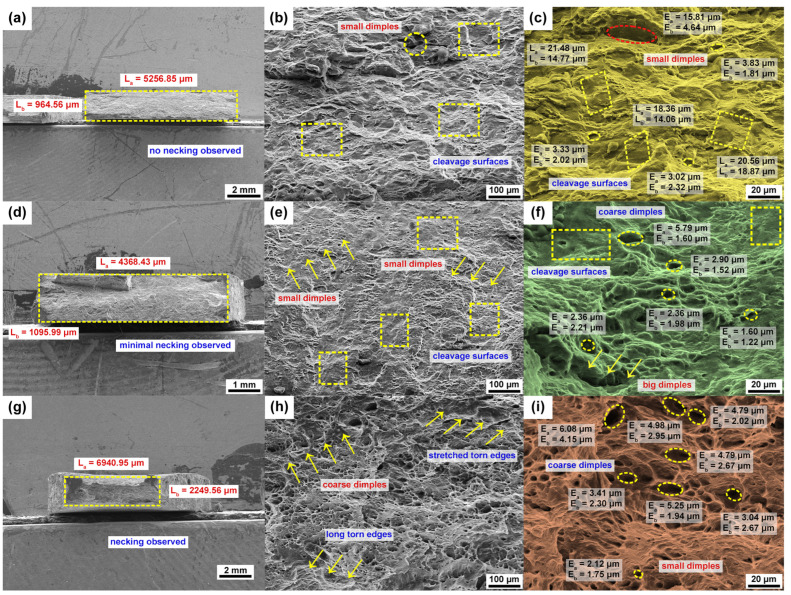
Microstructure images of AZ31B magnesium alloy by using FESEM observation at various magnifications. (**a**–**c**) 25 °C, (**d**–**f**) 100 °C, (**g**–**i**) 200 °C at 0.0167 s${}^{-1}$ in 90° rolling direction.

Additionally, the metallographic microstructure on the sample surface was observed via optical microscope (OM), as depicted in Figure 3 [26]. Furthermore, a field emission scanning electron microscopy (FESEM) (MIRA3 TESCAN, secondary electron detector, Seoul national university of science and technology, Seoul, South Korea) [27,28] was used to examine the tested samples for reviewing the fractured morphology during warm tensile deformation, as depicted in Figure 4.

## 3. Results and Discussion

### 3.1. AZ31B Magnesium Alloy Deformation Behavior

The representative true flow curves of AZ31B magnesium alloy at different temperatures (50 to 250 °C) and strain rates (0.005 to 0.0167 s${}^{-1}$) with respect to three rolling directions, such as 0°, 45°, and 90°, are presented in Figure 2. As illustrated in Figure 2, temperatures and strain rates have meaningful impact on flow behavior. At lower temperature, the flow stress rises significantly at a specific strain rate, as depicted in Figure 2a–i, and on the contrary, as shown in Figure 2a–i, the flow stress also declines with the increment in the temperature at a specific strain rate. The flow stress curves for different temperatures and strain rates reveal distinct patterns. At certain strain rates (0.005 to 0.0167 s${}^{-1}$), flow stress curves exhibit a maximum stress within a significant strain range due to pronounced strain hardening, followed by a gradual decrease caused by softening behavior. However, at a 250 °C temperature, considering strain rates (0.005 to 0.0167 s${}^{-1}$), the flow stress curve shows an initial maximum stress at a minimal strain range due to initial strain hardening, followed by a steady-state stress decrease attributed to dynamic softening [29]. Higher deformation temperatures promote dynamic recrystallization (DRX) by facilitating grain boundary movement, which aids the DRX grains nucleation and growth and then dislocation annihilation over an extended time. Consequently, the stress level decreases [29]. The corresponding microstructures of the AZ31B magnesium alloy at 25 °C and 250 °C are illustrated in Figure 3. Figure 3a reveals that the initial microstructure can be observed to have twinning with a 16.40 μm grain size, and noticeably, we can also see that the surface can be observed to have both coarse and finer grains. On the other hand, in Figure 3b, it is more evident that at a 250 °C deformation temperature, the surface can be observed to have more newly recrystallized grains at a 30 μm scale, and moreover, we can notice much more finer grains on the scanned surface than Figure 3a as well. Moreover, the estimated average grain size at a 250 °C deformation temperature with the value of 13.63 μm also confirms that recrystallization occurred.

To further understand the material deformation under hot deformation, we studied the fractured surface morphology using the FESEM technique, as represented in Figure 4. Figure 4 reveals the microstructure images of the fractured specimens for the 90° rolling direction at various magnification scales under various deformation conditions. As shown in Figure 4a, the surface morphology was observed at the fractured surface under a 25 °C temperature and 0.0167 s${}^{-1}$ strain rate. As can be observed in Figure 4a at a 2 mm scale, it was identified that necking cannot be visibly observed at room temperature. This observation confirms why the AZ31B magnesium alloy material reveals poor material formability (25 °C), as the material can be noticed to have a possibly brittle fracture. To confirm this statement, the surface was scanned at a higher (100 μm) scale, and Figure 4b, at a 100 μm scale, reveals that the scanned surface has more cleavage platforms, small dimples, and short torn edges, which indicates the poor material plasticity. Additionally, the surface was scanned at a 20 μm scale, and the quantification of cleavage platforms and dimples were made to confirm the hypothesis, as shown in Figure 4c. Comparably, at a 100 °C deformation temperature with same strain rate, similar microstructures were made from the tested samples. Figure 4d at a 1 mm scale confirms that under a 100 °C deformation temperature, the fracture surface can be observed to have minimal necking and explains that the material changes from quasi-cleavage to ductile gradually. Moreover, Figure 4e, at a 100 μm, scale provides more information about how cleavage platforms slowly change into a smaller size, and Figure 4f, at a 20 μm, scale explains how small dimples start to appear in most of the fracture surfaces under a higher deformation temperature. However, Figure 4d–f explain that the material formability is not improved under a 100 °C deformation temperature. Likewise, under a 200 °C deformation temperature with same strain rate, comparable microstructures were made, as shown in Figure 4g–i. Figure 4g at a 2 mm scale shows that as a result of ductile fracture, necking can be clearly observed and indicates that the material has undergone a large degree of plastic deformation before cracking confirms the high material plasticity. Moreover, Figure 4h at a 100 μm scale shows coarse dimples and long torn edges, which indicates material ductile fracture. As shown in Figure 4i at a 20 μm scale, it is obvious that an increase in the dimple size is due to grain refinement as well as material softening. A comparison of Figure 4g and Figure 4a,d explains that AZ31B magnesium alloy material formability is heightened under a 200 °C deformation temperature when compared to room temperature.

Overall, for rolling directions such as 0°, 45°, and 90°, the flow stress of the experimental AZ31B magnesium alloy rises with a decline in temperature and an increment in strain rate. The true stress curves presented in Figure 2a–i demonstrate that the initial deformation results in work hardening, followed by dynamic softening due to dynamic recovery and dynamic re-crystallization processes. Additionally, the dynamic softening effect is more pronounced at larger temperatures and smaller strain rates, as it is influenced by increased grain boundary mobility and extended nucleation and grain growth time [24]. It is worth noting that AZ31B magnesium alloy flow stress is highly responsive to strain rates at higher temperatures compared to lower temperatures [1].

### 3.2. Development of Constitutive Models

#### 3.2.1. Johnson–Cook Model

The JC material model is a popular semi-empirical constitutive model used to describe the plastic behavior of materials under high strains, strain rates, and temperatures [30]. Its simplicity, straightforward formulation, and ease of estimating material constants have made it used by researchers to forecast material flow behavior [30]. The JC model can be represented as [30,31,32,33]:

(1) $$\sigma =\left(A+B\varepsilon^{n}\right)\left[1+C\;\ln\left(\frac{\dot{\varepsilon }}{{\dot{\varepsilon }}_{r}}\right)\right]\left[1-{\left(\frac{T-T_{r}}{T_{m}-T_{r}}\right)}^{m}\right],$$

where *A*, *B*, *n*, *C*, *T*, and *m* are the model coefficients [27,28,29,33]. Equation (1) [30] represents the elasto-plastic term, which shows the work hardening effect and viscosity term, which reveals strain-rate-strengthening effect and thermal softening term, which reveals the temperature effect that influences the material flow stress [34,35]. Here, the melting temperature ($T_{m}$), the reference temperature ($T_{r}$), and the reference strain rate (${\dot{\varepsilon }}_{r}$) were assumed as 630 °C, 50 °C, and 0.005 s${}^{-1}$, respectively. For example, for a 0° rolling direction (RD), the yield stress *A* under the reference deformation conditions was determined as 186.163 MPa.

*Determination of Material Constants*: At reference conditions such as 50 °C and 0.005 s${}^{-1}$, Equation (1) can be altered into Equation (2) [10,11,12]:

(2) $$\sigma =(A+B\varepsilon^{n}).$$

Applying natural logarithms in Equation (2) delivers Equation (3) as shown below [10,11,12]:

(3) ln(σ−A)=nlnε+lnB.

**Figure 5:**
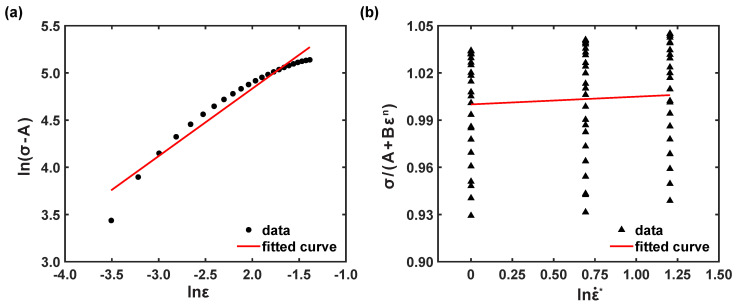
Correlation plots under reference conditions (**a**) ln(σ−A) vs. lnε and (**b**) $\frac{\sigma }{\left(A+B\varepsilon^{n}\right)}$ vs. $\ln{\dot{\varepsilon }}^{*}$.

A correlation plot of ln(σ−A) vs. lnε was outlined, as depicted in Figure 5a. Thus, *B* and *n* were calculated as 536.359 MPa and 0.719, respectively.

Equation (1) can be rearranged, when *T* is $T_{r}$ and expressed as Equation (4) [10,11,12].

(4) $$\frac{\sigma }{\left(A+B\varepsilon^{n}\right)}=\left(1+C\;\ln{\dot{\varepsilon }}^{*}\right).$$

By substituting, the material coefficients, like *A*, *B*, and *n*, and corresponding stress values at reference conditions, $\frac{\sigma }{\left(A+B\varepsilon^{n}\right)}∼\ln{\dot{\varepsilon }}^{*}$, were drawn, as represented in Figure 5b. Then, the slope was obtained from the fitted curve as *C*, and *C* was estimated as 0.0049.

Similarly, Equation (1) can be simplified, when $\dot{\varepsilon }$ is ${\dot{\varepsilon }}_{r}$ as [36,37,38,39]:

(5) $$\sigma =\left(A+B\varepsilon^{n}\right)\left[1-{\left(\frac{T-T_{r}}{T_{m}-T_{r}}\right)}^{m}\right].$$

Equation (6) can be received by applying the natural logarithm of Equation (5) as [36,37,38,39]

(6) $$\ln\left[1-\frac{\sigma }{\left(A+B\varepsilon^{n}\right)}\right]=m\;\ln T^{*}.$$

**Figure 6:**
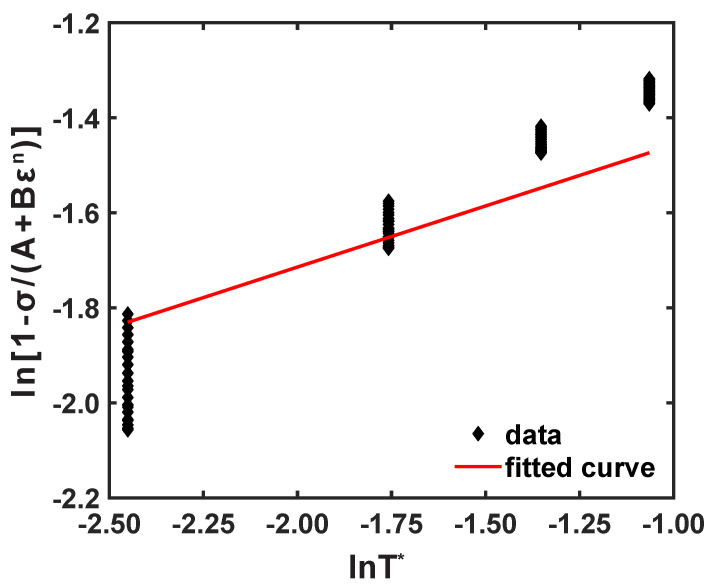
Correlation of $\ln\left[1-\frac{\sigma }{\left(A+B\varepsilon^{n}\right)}\right]$ vs. $\ln T^{*}$ under reference conditions.

By substituting, the material coefficients, like *A*, *B*, and *n*, and corresponding stress values at reference conditions, $\ln\left[1-\frac{\sigma }{\left(A+B\varepsilon^{n}\right)}\right]∼\ln T^{*}$, were plotted, as shown in Figure 6, and *m* was obtained as 0.643. In conclusion, the JC flow stress model of AZ31B magnesium alloy material for a 0° rolling direction can be established as follows:

(7) $${\hat{\sigma }}_{\mathrm{pred}}=\left(186.163+536.359\varepsilon^{0.719}\right)\left[1+0.0049\;\ln\left(\frac{\dot{\varepsilon }}{{\dot{\varepsilon }}_{r}}\right)\right]\left[1-{\left(\frac{T-T_{r}}{T_{m}-T_{r}}\right)}^{0.662}\right].$$

#### 3.2.2. Modified Johnson–Cook Model

The modified JC model can also be used to describe the material deformation behavior of the AZ31B magnesium alloy, and it can be represented as [18,25]:

(8) $$\sigma =\left(A_{1}+B_{1}\varepsilon +B_{2}\varepsilon^{2}\right)\left[1+C_{1}\;\ln\left(\frac{\dot{\varepsilon }}{{\dot{\varepsilon }}_{r}}\right)\right]\exp\left[\left(\lambda_{1}+\lambda_{2}\;\ln\left(\frac{\dot{\varepsilon }}{{\dot{\varepsilon }}_{r}}\right)\right)\left(T-T_{r}\right)\right],$$

where $A_{1}$, $B_{1}$, $B_{2}$, $C_{1}$, $\lambda_{1}$, and $\lambda_{2}$ are the model coefficients. Here, $T_{r}$ and ${\dot{\varepsilon }}_{r}$ are assumed as 50°, and 0.005 s${}^{-1}$, respectively.

*Determination of constants*: For example, for a 0° RD, under the reference conditions 50 °C and 0.005 s${}^{-1}$, Equation (8) can be altered into Equation (9) [18,25]:

(9) $$\sigma =(A_{1}+B_{1}\varepsilon +B_{2}\varepsilon^{2}).$$

**Figure 7:**
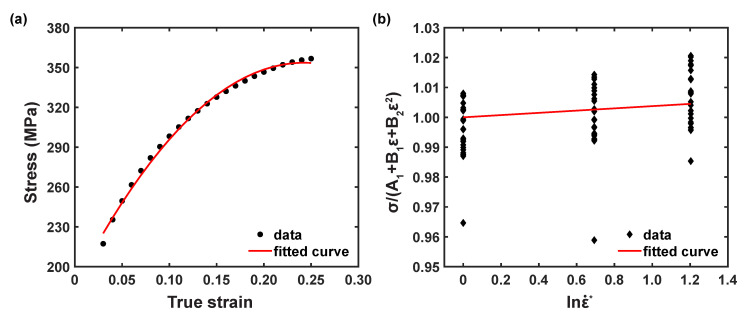
Correlation plots (**a**) σ vs. ε at reference conditions and (**b**) $\sigma /\left(A_{1}+B_{1}\varepsilon +B_{2}\varepsilon^{2}\right)$ vs. $\ln{\dot{\varepsilon }}^{*}$.

As revealed in Figure 7a, under the reference conditions, the relationship plot σ and ε was plotted using the second order polynomial equation, and the material parameters, such as $A_{1}$, $B_{1}$, and $B_{2}$, can be estimated as 185.235 MPa, 1422.22 MPa, and −2987.32 MPa, respectively, from the coefficients of the fitted polynomial equation.

Under the reference temperature condition, Equation (8) can be modified and rewritten as Equation (10) [18,25]:

(10) $$\frac{\sigma }{\left(A_{1}+B_{1}\varepsilon +B_{2}\varepsilon^{2}\right)}=\left[1+C_{1}\;\ln\left(\frac{\dot{\varepsilon }}{{\dot{\varepsilon }}_{r}}\right)\right].$$

By substituting the estimated material constants, considering the stress values from the tested strain rate conditions at $T_{r}$, the relationship plot between the dimensionless strain rate, $\ln{\dot{\varepsilon }}^{*}$ and $\frac{\sigma }{\left(A_{1}+B_{1}\varepsilon +B_{2}\varepsilon^{2}\right)}$, was plotted, as illustrated in Figure 7b. Thus, the material coefficient, $C_{1}$, was computed as 0.0037.

For the tested conditions, Equation (8) can be rearranged and written as Equation (11) [18,25]:

(11) $$\frac{\sigma }{\left(A_{1}+B_{1}\varepsilon +B_{2}\varepsilon^{2}\right)\left(1+C_{1}\;\ln{\dot{\varepsilon }}^{*}\right)}=\exp\left[\left(\lambda_{1}+\lambda_{2}\;\ln{\dot{\varepsilon }}^{*}\right)T^{*}\right].$$

Applying natural logarithm in Equation (11) delivers Equation (12) as follows:

(12) $$\ln\left[\frac{\sigma }{\left(A_{1}+B_{1}\varepsilon +B_{2}\varepsilon^{2}\right)\left(1+C_{1}\;\ln{\dot{\varepsilon }}^{*}\right)}\right]=\left(\lambda_{1}+\lambda_{2}\;\ln{\dot{\varepsilon }}^{*}\right)T^{*}.$$

**Figure 8:**
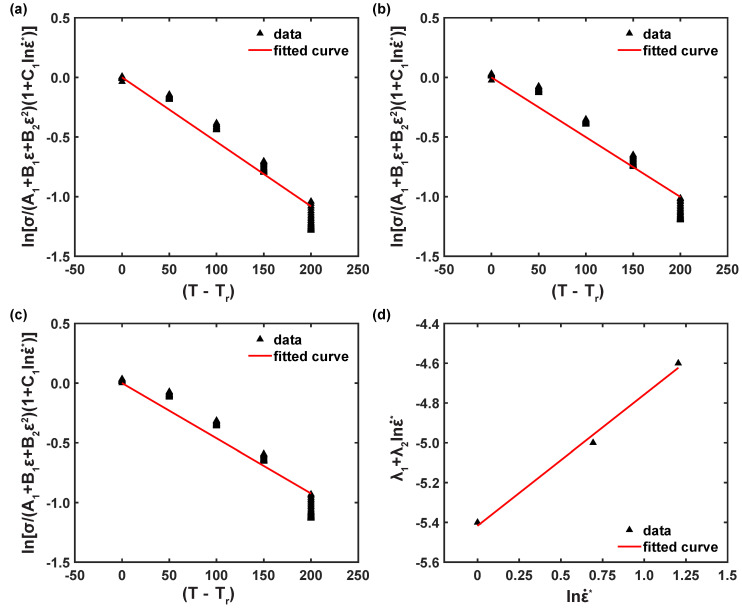
Correlation plots (**a**–**c**) $\ln[\sigma /\left(A_{1}+B_{1}\varepsilon +B_{2}\varepsilon^{2}\right)\left(1+C_{1}\;\ln{\dot{\varepsilon }}^{*}\right)]$ vs. $T^{*}$ and (**d**) λ vs. $\ln\dot{\varepsilon }$.

Equation (12) was simplified by adding a new coefficient, named λ, which is equal to $\left(\lambda_{1}+\lambda_{2}\;\ln\dot{\varepsilon }\right)$, and λ can be estimated from the correlation between $\ln\left[{}^{\sigma }\;{/}_{\left(A_{1}+B_{1}\varepsilon +B_{2}\varepsilon^{2}\right)\left(1+C_{1}\ln{\dot{\varepsilon }}^{*}\right)}\right]$ and $T^{*}$, as shown in Figure 8a. Furthermore, in this study, we considered three strain rates, so three graphs were plotted and the introduced parameter, λ, was derived from each plot, as depicted in Figure 8b,c. The introduced new parameter, λ, in Equation (12) can be presented as Equation (13) [18,25],

(13) $$\lambda =\lambda_{1}+\lambda_{2}\;\ln\dot{\varepsilon }.$$

Eventually, as revealed in Figure 8d, the model coefficients, $\lambda_{1}$ and $\lambda_{2}$, are calculated as −0.00542 and 0.000659, respectively. Thus, the modified JC flow stress model of the AZ31B magnesium alloy material for a 0° rolling direction can be established as follows:

$$\begin{array}{ccc} {\hat{\sigma }}_{\mathrm{pred}} & = & \left(185.235+1422.22\varepsilon -2987.32\varepsilon^{2}\right)\left(1+0.0037\;\ln\left(\frac{\dot{\varepsilon }}{{\dot{\varepsilon }}_{r}}\right)\right)\\ & \; & \;\;\exp\left[\left(-5.42\times 10^{-3}+6.59\times 10^{-4}\;\ln\left(\frac{\dot{\varepsilon }}{{\dot{\varepsilon }}_{r}}\right)\right)\left(T-T_{r}\right)\right]. \end{array}$$

#### 3.2.3. Modified Zerilli–Armstrong Model

The modified ZA model is also used to describe the material plastic deformation behavior of AZ31B magnesium alloy, and it can be represented as [18,25]:

(14) $$\sigma =\left(C_{1}+C_{2}\varepsilon^{n}\right)\exp\left[-\left(C_{3}+C_{4}\varepsilon \right)\left(T-T_{r}\right)+\left(C_{5}+C_{6}\left(T-T_{r}\right)\right)\ln\left(\frac{\dot{\varepsilon }}{{\dot{\varepsilon }}_{r}}\right)\right],$$

where $C_{1}$, $C_{2}$, *n*, $C_{3}$, $C_{4}$, $C_{5}$, and $C_{6}$ are the model coefficients. Here, $T_{r}$ and ${\dot{\varepsilon }}_{r}$ are considered as 50 °C and 0.005 s${}^{-1}$, respectively. For example, for 0° RD, the yield stress, $C_{1}$, under the reference deformation conditions was determined as 186.163 MPa.

*Determination of constants*: At ${\dot{\varepsilon }}_{r}$, Equation (14) can be rearranged and represented as Equation (15) [18,25]:

(15) $$\sigma =\left(C_{1}+C_{2}\varepsilon^{n}\right)\exp\left[-\left(C_{3}+C_{4}\varepsilon \right)T^{*}\right],$$

Then, by applying the natural logarithm in Equation (15) [25], Equation (16) [25] can be obtained as [18,25]:

(16) $$\ln\sigma =\ln\left(C_{1}+C_{2}\varepsilon^{n}\right)-\left(C_{3}+C_{4}\varepsilon \right)T^{*},$$

(17) $$I_{1}=\ln\left(C_{1}+C_{2}\varepsilon^{n}\right),$$

(18) $$s_{1}=-C_{3}+C_{4}\varepsilon ,$$

**Figure 9:**
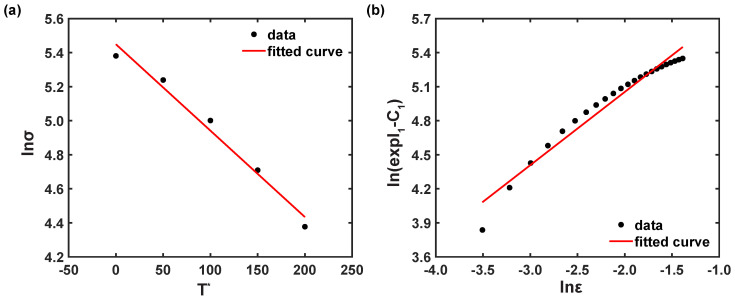
Correlation plots (**a**) lnσ vs. $T^{*}$ at ε = 0.3 and (**b**) $\ln(\exp\left(I_{1}\right)-C_{1})$ vs. lnε.

By putting flow stress at ${\dot{\varepsilon }}_{r}$ into Equation (16), $S_{1}$ and $I_{1}$ can be computed from lnσ vs. $T^{*}$, as illustrated in Figure 9a. The steps were repeated for other strain values, and then, Equation (19) [25] was received by applying the natural logarithm in Equation (17) as [18,25]:

(19) $$\ln\left(\exp\left(I_{1}\right)-C_{1}\right)=\ln C_{2}+n\ln\varepsilon .$$

At ${\dot{\varepsilon }}_{r}$, by adopting stress values from the entire temperature range and using the estimated values of $C_{1}$ and $I_{1}$, the correlation plot of $\ln(\exp\left(I_{1}\right)-C_{1})$ vs. lnε was achieved, as shown in Figure 9b. Thus, the model coefficients, $C_{2}$ and *n*, were determined as 569.1249 MPa and 0.6453, respectively, from the information of the fitted curve.

**Figure 10:**
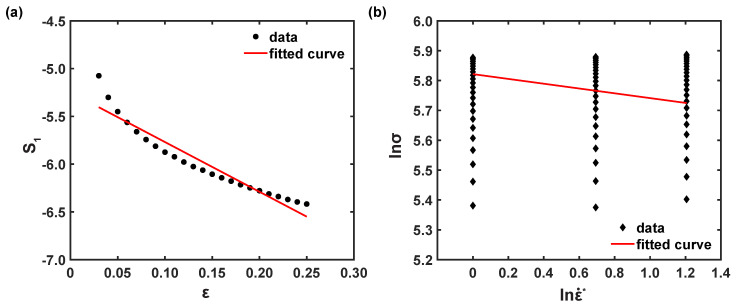
Correlations of (**a**) $S_{1}$ vs. ε and (**b**) lnσ vs. $\ln{\dot{\varepsilon }}^{*}$ at 50 °C.

Similar to the coefficients $C_{2}$ and *n*, at ${\dot{\varepsilon }}_{r}$, by substituting estimated $S_{1}$ into the discrete true strains, the coefficients $C_{3}$ and $C_{4}$ were computed as 0.0052 and 0.0052, respectively, from the linear model information of ε vs. $S_{1}$, as depicted in Figure 10a.

Applying the natural logarithm in Equation (14) delivers Equation (20) as follows [25]:

(20) $$\ln\sigma =\ln\left(C_{1}+C_{2}\varepsilon^{n}\right)-\left(C_{3}+C_{4}\varepsilon \right)T^{*}+\left(C_{5}+C_{6}T^{*}\right)\ln{\dot{\varepsilon }}^{*},$$

(21) $$S_{2}=C_{5}+C_{6}T^{*},$$

**Figure 11:**
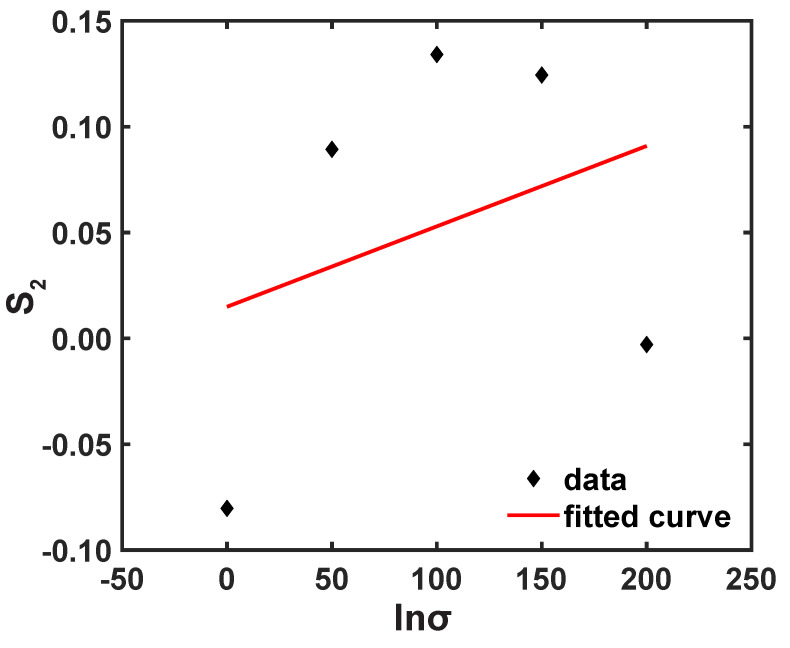
Correlation plot of $S_{2}$ vs. lnσ.

For accounted strain rates with respect to one temperature, the relationship plot of lnσ vs. $\ln{\dot{\varepsilon }}^{*}$ can be made, as illustrated in Figure 10b. Then, the coefficient, $S_{2}$, was estimated from Figure 10b at a specific temperature. For five temperatures, five different values of $S_{2}$ were determined, and thereafter, the parameters $C_{5}$ and $C_{6}$ were computed as 0.0149 and 0.0004, respectively, from the fitted curve information, as depicted in Figure 11. Thus, the modified ZA flow stress model of the AZ31B magnesium alloy material for 0° RD can be established as follows:

$$\begin{array}{ccc} {\hat{\sigma }}_{\mathrm{pred}} & = & \left(186.163+569.1249\varepsilon^{0.6453}\right)\\ & \; & \;\;\exp\left[-\left(0.0052+0.0052\varepsilon \right)T^{*}+\left(0.0149+0.0004T^{*}\right)\ln{\dot{\varepsilon }}^{*}\right]. \end{array}$$

**Figure 12:**
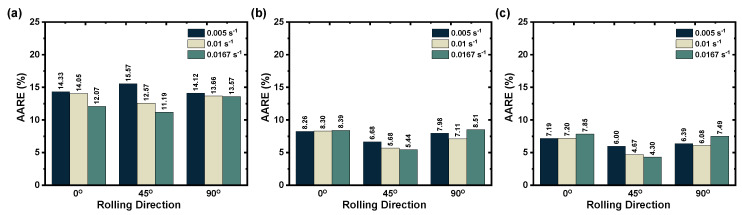
Prediction error of the proposed flow stress models. (**a**) original JC model; (**b**) modified JC model; (**c**) modified ZA model.

Using the estimated model parameters of the proposed constitutive models in Table 1, Table 2 and Table 3, AZ31B magnesium alloy flow stress data under the considered deformation conditions for three rolling directions were calculated. In order to assess the accuracy of the proposed flow stress models, the AARE can be computed by comparing the test data with the predicted data using the following equation [28,40,41,42]:

(22) $$\mathrm{AARE}=\frac{1}{n}\sum_{i=1}^{n}\left|\frac{\sigma_{e}^{i}-\sigma_{p}^{i}}{\sigma_{e}^{i}}\right|\times 100\%,$$

where $\sigma_{e}$, $\sigma_{p}$, and *n* are the experimental true stress, the predicted true stress, and the total number of true stress data, respectively. Subsequently, the prediction errors were estimated using Equation (22) and plotted in Figure 12. As shown in Figure 12a, the original JC model could not sufficiently represent AZ31B magnesium alloy deformation flow behavior, as it shows higher prediction errors ranging from 11.19% to 15.57% across all rolling directions and deformation conditions. On the other hand, Figure 12b,c demonstrate that the proposed modified JC and ZA models offer good prediction of flow stress values for the AZ31B magnesium alloy. Additionally, the prediction errors are estimated to be about 4.30% to 8.51% across all rolling directions and deformation conditions considering both the modified JC and ZA models. The prediction error comparison confirms that the prediction error is reduced by about 45.34% to 61.57% when compared against the minimum and maximum prediction error of the original JC model. To assess the predictive capability of the proposed flow stress models, a comparison plot was created depicting the predicted flow curves alongside the experimental data. This allows for a detailed discussion of the model’s accuracy with respect to each test condition.

**Figure 13:**
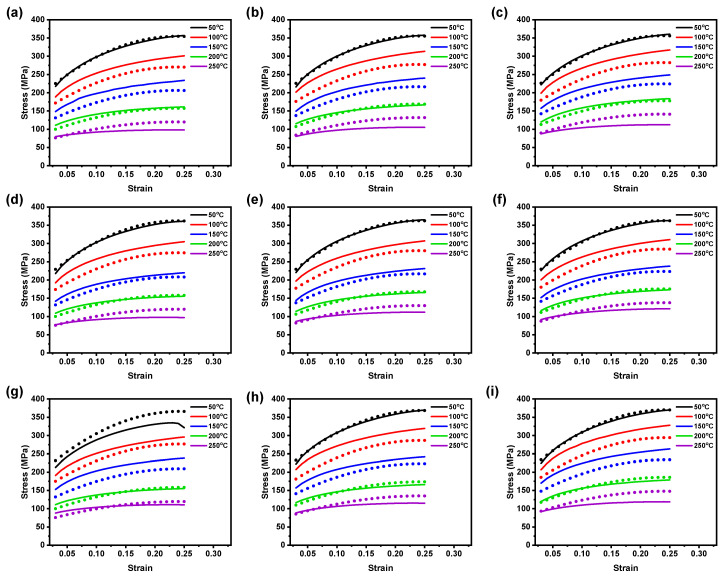
Comparison plot between test data (lines) vs. calculated data (dots) using the modified JC model at $\dot{\varepsilon }$ of (**a**) 0.005 s${}^{-1}$; (**b**) 0.01 s${}^{-1}$; (**c**) 0.0167 s${}^{-1}$ at 0° RD. (**d**) 0.005 s${}^{-1}$; (**e**) 0.01 s${}^{-1}$; (**f**) 0.0167 s${}^{-1}$ at 45° RD. (**g**) 0.005 s${}^{-1}$; (**h**) 0.01 s${}^{-1}$; (**i**) 0.0167 s${}^{-1}$ at 90° RD.

**Figure 14:**
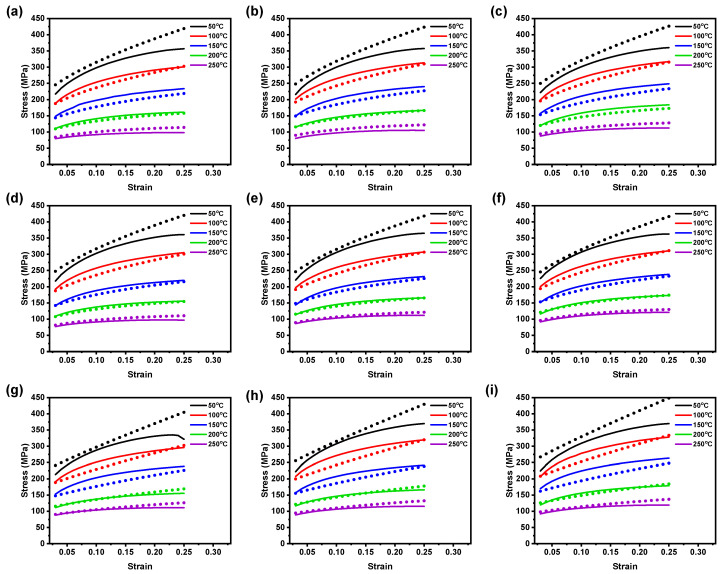
Comparison plot between test data (lines) vs. calculated data (dots) using the modified ZA model at $\dot{\varepsilon }$ of (**a**) 0.005 s${}^{-1}$; (**b**) 0.01 s${}^{-1}$; (**c**) 0.0167 s${}^{-1}$ at 0° RD. (**d**) 0.005 s${}^{-1}$; (**e**) 0.01 s${}^{-1}$; (**f**) 0.0167 s${}^{-1}$ at 45° RD. (**g**) 0.005 s${}^{-1}$; (**h**) 0.01 s${}^{-1}$; (**i**) 0.0167 s${}^{-1}$ at 90° RD.

According to Lin et al. [43], the original JC model’s predictability is constrained to a specific $T_{r}$ and ${\dot{\varepsilon }}_{r}$. This limitation arises from the model’s assumption on the coupled effects and independent phenomena. However, in practice, it is essential to account for the combined effects on the flow behavior of the AZ31B magnesium alloy [18,43,44]. A comparison between the test and calculated data from the modified JC and ZA models under the tested conditions is outlined in Figure 13 and Figure 14. Figure 13 and Figure 14 show good agreement at high temperatures, indicating that both the modified JC and modified ZA models accurately predict AZ31B magnesium alloy flow stress values. These models are suitable for analyzing hot deformation behavior in sheet-metal-forming processes. The modified ZA model demonstrates good accuracy in forecasting deformation behavior at elevated temperatures compared to the modified JC model, as revealed in Figure 13 and Figure 14. In detail, as depicted in Figure 13, the recognized modified JC model showed good prediction against the experimental observations under the reference conditions (50 to 250 °C and 0.005 to 0.0167 s${}^{-1}$). Moreover, at 200 °C for the tested strain rates across all the rolling directions, the model provided better predictions against the test data. Similar observations were also made for 150 °C and 250 °C temperatures for the considered strain rates; however, there were some noticeable deviations in the predicted data.

Compared with the other test conditions, Figure 13 reveals that the established modified JC model could not significantly forecast the material deformation behavior at 100 °C for the entirety of the test conditions. Subsequently, as shown in Figure 14a–f, the calculated flow stress data from the modified ZA model falls close to the test observations at 100 °C to 250 °C at the tested strain rates for 0° and 45° RDs; however, for 90° RD, the proposed MZA model could represent the material flow behavior at only 200 °C and 250 °C, including reported strain rates, as depicted in Figure 14. Furthermore, under 50 °C and 100 °C test conditions, high prediction deviations were observed, as shown in Figure 14. In addition, for three rolling directions, the prediction errors were observed to be higher than the other test conditions. Thus, the modified ZA model outperforms the original JC and modified JC models in accurately representing AZ31B magnesium alloy deformation behavior across the considered processing conditions, as outlined in Figure 14. The improved performance of the modified ZA model can be attributed to the combined effects of deformation temperature and strain rate on the flow stress.

The proposed constitutive equation predictability can be further verified through employing statistical metrics, like $R^{2}$ and RMSE, as follows [28,40,41,42]:

(23) $$R^{2}=1-\frac{\sum_{i=1}^{n}{\left(\sigma_{e}^{i}-\sigma_{p}^{i}\right)}^{2}}{\sum_{i=1}^{n}{\left(\sigma_{e}^{i}-{\bar{\sigma }}_{e}\right)}^{2}},$$

(24) $$\mathrm{RMSE}=\sqrt{\frac{1}{n}\sum_{i=1}^{n}{\left(\sigma_{e}^{i}-\sigma_{p}^{i}\right)}^{2}}$$

**Figure 15:**
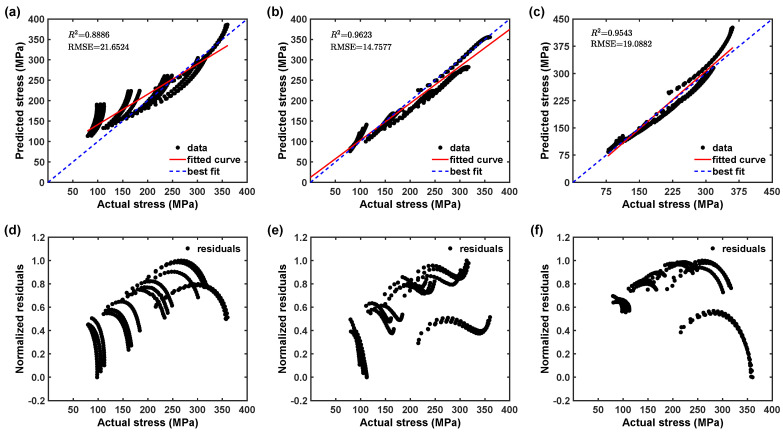
Correlation plots from 0° RD (**a**) original JC, (**b**) modified JC, (**c**) modified ZA models. Residual plots (**d**), original JC (**e**), modified JC (**f**), modified ZA models.

**Figure 16:**
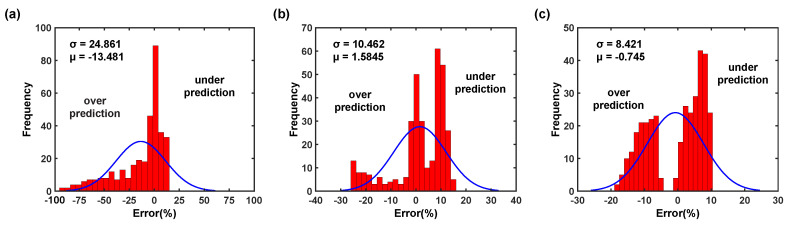
Histogram plots from 0° RD (**a**) original JC, (**b**) modified JC, (**c**) modified ZA models.

The $R^{2}$ measures the linear relationship strength, while RMSE provides information on the comparison of relative errors term by term. Figure 15a reveals that the calculated flow stress data deviate from the best-fit line, with an estimated $R^{2}$ value of 0.888. This suggests that the original JC model fails to accurately capture the material’s deformation behavior. Furthermore, Figure 15d also demonstrates that the residual distribution is not random, indicating that the model lacks predictability due to missing terms in the constitutive equation. Moreover, Figure 16a shows that the original JC model considerably underestimates the flow stress, which results in high prediction error. In contrast, Figure 15b illustrates that the modified JC model yields predicted data that closely align with the best-fit line, with a correlation coefficient of 0.962, which is significantly higher than the original JC model. This signifies an improved correlation between the predicted and test data. However, despite the improvement in prediction, Figure 15e reveals that the residual distribution is still non-random, indicating that the modified JC model also overlooks certain terms in the constitutive equation, resulting in remaining prediction errors.

Additionally, Figure 16b displays that the modified JC model showed the prediction error reduction with high range of under predictions (within ±30%) of the flow stress. Similarly, as shown in Figure 15c, the predicted data mostly fall near the best-fit line for the modified ZA model, as well with an $R^{2}$ of 0.954, which is significantly higher than the original JC model. This also explains that the modified ZA model has better predictability than the original JC model. However, Figure 15f demonstrates that the residual distribution is still not random and explains that the modified ZA model also misses some terms in the constitutive equation, which might be the reason for the remaining prediction error. Additionally, Figure 16c displays that the modified ZA model showed a prediction error reduction somehow balanced in the range of under and over predictions (within ±20%). The predictability of the original JC, modified JC, and modified ZA models is summarized in Table 4 and Table 5. Based on the discussion above, it is evident that the proposed modified JC and ZA models provide good predictions for the flow stress values of the AZ31B magnesium alloy. These models are suitable and reliable for analyzing the AZ31B magnesium alloy hot deformation process.

## 4. Conclusions

Extensive investigations were conducted on the hot deformation behavior of an AZ31B magnesium alloy over a broad range of temperatures and strain rates. The objective was to identify an appropriate constitutive equation that accurately captures the material’s deformation behavior, utilizing warm tensile test data. The following can be concluded:

1. Increasing strain rates and decreasing deformation temperatures result in higher flow stress in the AZ31B magnesium alloy. The material demonstrates greater sensitivity to strain rates at higher temperatures than lower temperatures. Additionally, at elevated temperatures, dynamic recrystallization occurs, leading to finer grains on the surface compared to room temperature.
2. The original JC model was identified to be inadequate to provide a good description of flow behavior. This is because the original JC model does not consider the coupled effects of strain, strain rates, and temperatures. For example, the numerical quantification, such as $R^{2}$, 0.888; AARE, 13.483%, from 0° RD also confirms that the original JC model could not forecast the flow behavior effectively.
3. The modified JC model showed good capability to represent the material flow behavior of the AZ31B magnesium alloy under the reference conditions at higher temperatures. Moreover, the estimated statistical metrics, such as $R^{2}$, 0.962; AARE, 8.318%, from 0° RD indicate that the modified JC model can forecast the material flow behavior better than the original JC model. The statistical parameter also explains that the modified JC model has a good correlation against test data comparison when compared to the original JC model.
4. Similarly, the modified ZA model also revealed better capability to characterize the material flow behavior of the AZ31B magnesium alloy at higher temperatures with the considered strain rates. Additionally, the estimated parameters, $R^{2}$, 0.954; AARE, 7.413%, from 0° RD demonstrate that the modified ZA model can characterize the material flow behavior with higher accuracy than the original JC model. The numerical quantification also clarifies that the modified ZA model has a good correlation against test data comparison when compared to the original JC model.

In summary, the comparison of experimental and predicted data for the original JC model, modified JC model, and modified ZA model suggests that the modified JC and modified ZA models are effective in predicting the tensile flow behavior of an AZ31B magnesium alloy under hot deformation conditions, offering good prediction accuracy.

## Figures and Tables

**Table 1:** JC material model parameters of AZ31B magnesium alloy.

Angle	Original JC Model				
	*A* (MPa)	*B* (MPa)	*n*	*C*	*m*
**0°**	186.163	536.359	0.719	0.0049	0.643
**45°**	187.871	541.828	0.707	0.0022	0.613
**90°**	211.682	799.457	1.005	0.0032	0.607

**Table 2:** Modified JC model coefficients of AZ31B magnesium alloy.

Angle	Modified JC Model					
	*A*${}_{1}$ (MPa)	*B* ${}_{1}$	B${}_{2}$	*C* ${}_{1}$	λ ${}_{1}$	λ ${}_{2}$
**0°**	185.235	1422.22	−2987.32	0.0037	−$5.42\times 10^{-3}$	$6.59\times 10^{-4}$
**45°**	188.238	1457.03	−3054.24	0.0011	−$5.53\times 10^{-3}$	$5.73\times 10^{-4}$
**90°**	190.794	1439.26	−2955.42	0.0092	−$5.59\times 10^{-3}$	$8.33\times 10^{-4}$

**Table 3:** Modified ZA model coefficients of AZ31B magnesium alloy.

Angle	Modified ZA Model						
	*C* ${}_{1}$	*C* ${}_{2}$	*n*	*C* ${}_{3}$	*C* ${}_{4}$	*C* ${}_{5}$	*C* ${}_{6}$
**0°**	186.163	569.1249	0.6453	0.0052	0.0052	0.0149	$4.00\times 10^{-4}$
**45°**	187.871	564.1386	0.6405	0.0054	0.0051	−0.0074	$7.09\times 10^{-4}$
**90°**	211.682	662.7514	0.8908	0.0048	0.0042	0.0854	−$8.90\times 10^{-5}$

**Table 4:** Statistical measurements of proposed constitutive models.

Angle	Conditions	JC Model		Modified JC Model		Modified ZA Model	
		$R^{2}$	Overall $R^{2}$	$R^{2}$	Overall $R^{2}$	$R^{2}$	Overall $R^{2}$
**0°**	**0.005 s^−1^**	0.8954	0.8886	0.9679	0.9623	0.9611	0.9543
	**0.01 s^−1^**	0.8866		0.9607		0.9526	
	**0.0167 s^−1^**	0.8919		0.9590		0.9491	
**45°**	**0.005 s^−1^**	0.8994	0.9053	0.9751	0.9783	0.9698	0.9736
	**0.01 s^−1^**	0.9132		0.9805		0.9760	
	**0.0167 s^−1^**	0.9143		0.9795		0.9752	
**90°**	**0.005 s^−1^**	0.8550	0.8501	0.9539	0.9519	0.9454	0.9470
	**0.01 s^−1^**	0.8680		0.9664		0.9589	
	**0.0167 s^−1^**	0.8549		0.9500		0.9372	

**Table 5:** Estimated prediction error from the proposed constitutive models.

Angle	AARE (%)		
	JC Model	Modified JC Model	Modified ZA Model
**0°**	13.483	8.318	7.413
**45°**	13.109	5.934	4.991
**90°**	13.786	7.867	6.653

## Data Availability

Not applicable.

## Abbreviations

The following abbreviations are used in this manuscript:

JC	Johnson–Cook
ZA	Zerilli–Armstrong
FESEM	Field-emission scanning electron microscopy
FE	Finite element
$R^{2}$	Coefficient of determination
AARE	Average absolute relative error
RMSE	Relative mean square error
